# Fabrication and Thermo-Electro and Mechanical Properties Evaluation of Helical Multiwall Carbon Nanotube-Carbon Fiber/Epoxy Composite Laminates

**DOI:** 10.3390/polym13091437

**Published:** 2021-04-29

**Authors:** Alamry Ali, Andri Andriyana, Shukur Bin Abu Hassan, Bee Chin Ang

**Affiliations:** 1 Department of Mechanical Engineering, Faculty of Engineering, Universiti Malaya, Kuala Lumpur 50603, Malaysia; a.alamry@psau.edu.sa; 2 Center of Advanced Materials, Faculty of Engineering, Universiti Malaya, Kuala Lumpur 50603, Malaysia; amelynang@um.edu.my; 3 Centre for Advanced Composite Materials (CACM), Universiti Teknologi Malaysia, Skudai 81310, Malaysia; shukur@utm.my; 4 School of Mechanical Engineering, Faculty of Engineering, Universiti Teknologi Malaysia, Skudai 81310, Malaysia; 5 Department of Chemical Engineering, Faculty of Engineering, Universiti Malaya, Kuala Lumpur 50603, Malaysia

**Keywords:** helical multiwalled carbon nanotubes (HMWCNTs), composite laminate, interfacial bond, fracture toughness, dispersion technique

## Abstract

The development of advanced composite materials has taken center stage because of its advantages over traditional materials. Recently, carbon-based advanced additives have shown promising results in the development of advanced polymer composites. The inter- and intra-laminar fracture toughness in modes I and II, along with the thermal and electrical conductivities, were investigated. The HMWCNTs/epoxy composite was prepared using a multi-dispersion method, followed by uniform coating at the mid-layers of the CF/E prepregs interface using the spray coating technique. Analysis methods, such as double cantilever beam (DCB) and end notched flexure (ENF) tests, were carried out to study the mode I and II fracture toughness. The surface morphology of the composite was analyzed using field emission scanning electron microscopy (FESEM). The DCB test showed that the fracture toughness of the 0.2 wt.% and 0.4 wt.% HMWCNT composite laminates was improved by 39.15% and 115.05%, respectively, compared with the control sample. Furthermore, the ENF test showed that the mode II interlaminar fracture toughness for the composite laminate increased by 50.88% and 190%, respectively. The FESEM morphology results confirmed the HMWCNTs bridging at the fracture zones of the CF/E composite and the improved interlaminar fracture toughness. The thermogravimetric analysis (TGA) results demonstrated a strong intermolecular bonding between the epoxy and HMWCNTs, resulting in an improved thermal stability. Moreover, the differential scanning calorimetry (DSC) results confirmed that the addition of HMWCNT shifted the *T*_g_ to a higher temperature. An electrical conductivity study demonstrated that a higher CNT concentration in the composite laminate resulted in a higher conductivity improvement. This study confirmed that the demonstrated dispersion technique could create composite laminates with a strong interfacial bond interaction between the epoxy and HMWCNT, and thus improve their properties.

## 1. Introduction

Polymer composite materials developed using advanced carbon-based additives are becoming increasingly popular because of their superior mechanical properties. Presently, a wide range of carbon-based additives are available to formulate composite materials for various applications. Carbon fiber reinforced polymer (CFRP) composites have numerous uses in application fields such as aerospace, marine, automotive, and infrastructure industries, because of their lower structural weight and enhanced mechanical properties, including specific stiffness, specific strength, and tensile modulus [1,2,3,4]. These materials possess excellent tension–tension fatigue resistance, impact resistance, corrosion resistance, durability. and in-plane tensile strength [5,6,7,8]. However, there are some drawbacks in the properties of such fiber reinforced plastics (FRPs). These setbacks seriously affect the overall performance of a composite, and include low through-thickness properties, brittle matrix, and weak intermolecular bonds at the fiber–matrix interface [9,10]. The delamination growth behavior of composite laminates has been investigated and quantified experimentally through the Paris crack growth equation and the strain energy release rate [11,12,13]. The peak interlaminar fatigue crack growth behavior of the carbon fiber reinforced composite laminates subjected to mode I loading revealed matrix cracking and interface delamination as the dominant damage mechanisms [11,14,15]. Damage of the lamina and/or multiple failure phenomena in the laminated composites remain the leading problems to be solved. Furthermore, interlaminar delamination is one of the most significant challenges in the design of any structural composite [7]; others include a large number of design variables, problems related to the topology optimization of composite structures, specific non-linear behaviors of laminated structures, and uncertainties on the mechanical properties of composites, among others [7,16,17]. Brittle fractures originate in the matrix-dominated interlaminar area from fiber–matrix interface de-bonding and transverse matrix cracking, which rigorously influence the composite laminate structural integrity [18,19]. The source of such delamination between the composite layers is due to imperfections (e.g., micro-cracks) caused during the fabrication process. Such micro-cracks start to propagate in regions where comparatively higher void proportions exist under fatigue, impact stresses, and transverse loading conditions, or from different environmental factors [19,20,21]. On the other hand, FE simulations have been used to determine the interlaminar and matrix cracking failure process in HMWCNT-laden composite laminates. The cohesive strength of the interface has been predicted based on the periodic representative volume element (RVE) approach, which is incorporated into the cohesive zone model [11]. CZM has been used extensively to predict the strength of adhesive interface [7,22,23,24,25]. The results show that ductile adhesive joints are highly influenced by the CZM shape, and that the trapezoidal shape is best suited for the experimental validation. However, the high cost of simulation limits the adoption of this approach [12]. Many studies already published in the literature deal with these interlaminar interface deficiencies and imperfections. Several composite laminates designs have been reported to increase the strength and toughness, including 3D weaving [26,27], stitching [28,29], and Z-pinning [30]. Some of these processes decrease the in-plane laminate properties because of the damage produced by the insertion of reinforcement in the direction of thickness, with diameter in microns and volume fraction loss of micro-fiber in the in-plane direction [31]. Other methods to increase interlaminar toughness and matrix property modification include the addition of reinforced interlayers, toughening agents [32,33] or particles [34], and nanofibers [35], particularly carbon nanotubes (CNTs) [36,37,38], which have an excellent specific strength, stiffness, and aspect ratio [39,40]. Perhaps a great application of CNTs as nanofillers is in aerospace applications, because of their high electrical and thermal conductivity and other multifunctional properties [41,42,43,44,45]. It has also been reported and confirmed that the CNT possesses a 100 times greater strength than steel and 6 times less density [39,46,47]. Moreover, CNT incorporated carbon fiber has a high shear strength [48,49,50,51], interlaminar fracture toughness [19,52,53], and possesses a better reinforcement effect with matrix polymers [53,54,55,56]. Furthermore, CNT incorporated into a polymer matrix before infiltration to reduce the impact on the matrix viscosity during a small amount of loading has been reported in the literature [57,58,59,60]. A considerable enhancement was observed in the flexural strength, ultimate tensile strength, fracture toughness, and shear stress because of the combined MWCNT and graphite nanoplatelet (GNP) mechanical properties [61,62,63,64,65]. The impetus of this work is the urge to eliminate persistent interlaminar failure and inconsistent property regimes in the helical multiwalled carbon nanotubes (HMWCNTs) incorporated with carbon fiber epoxy (CF/E) composite laminates. Furthermore, this study serves to provide an experimental basis to the numerous FE simulation approaches used in tackling composite laminate delamination and matrix cracking failure processes. As such, further research, development, and investigations were necessary to formulate improved composite laminates and to understand the various underlying failure mechanisms.

The objective of this study is to determine the effect of varying the wt.% of HMWCNTs on the fracture strength, microstructure, and electrical and thermal properties of HMWCNT CF/E laminates. The focus is on the formulation, development, and characterization of HMWCNTs that are incorporated into CYCOM 934 unidirectional CF/E composite laminates. More specifically, HMWCNTs of unidirectional CF/E composite laminates were fabricated and their interlaminar fracture toughness in modes I and II was investigated. The carbon nanostructure CF/E laminates were fabricated using multi-dispersion techniques with varying wt.% of HMWCNTs loadings into the interface layer of prepregs. The fracture tests of different modes I and II were conducted using double cantilever beam (DCB) and end notched flexure (ENF) tests. The fracture surface morphology was evaluated using FESEM. Finally, the resulting thermal and electrical properties of the composite laminates were investigated.

## 2. Materials and Methods

### 2.1. Materials

**Figure 1:**
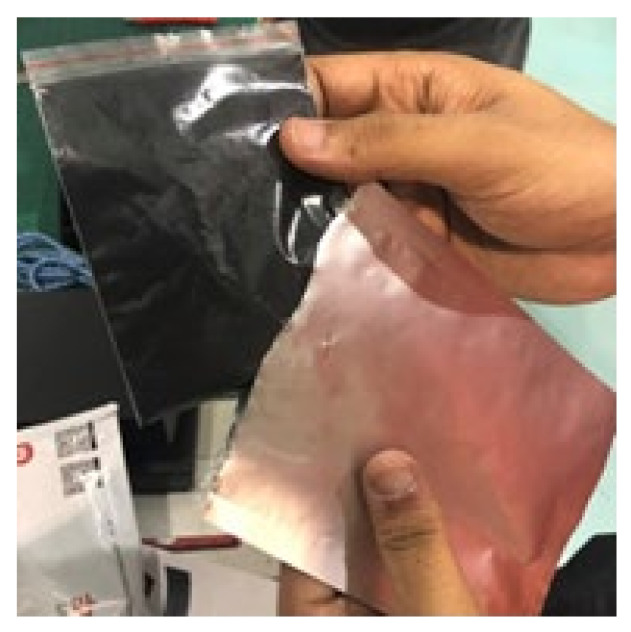
Helical multiwalled carbon nanotubes (HMWCNTs) used in the experimental work.

The EpoxyAmite 100 resin with 103 hardeners used in this work was supplied from (Smooth-on Inc., Macungie, PA, USA), while the HMWCNTs were purchased from (Cheap Tubes Inc., Grafton, VT, USA) (see Figure 1). The helical structure in the CNTs constituted 80 wt.% in fraction and the rest were standard CNTs with an outer diameter ranging 100–200 nm. CYCOM 934 unidirectional carbon fiber epoxy prepregs were used in this study.

### 2.2. Dispersion of Epoxy and HMWCNTs

The HMWCNTs/epoxy dispersion was prepared with the addition of 0.2 wt.% and 0.4 wt.% of HMWCNTs interleaves. To achieve a better dispersion quality, mechanical mixer, magnetic stirrer, and ultrasonication methods were used. A certain amount of HMWCNT was initially mixed with ethanol using a magnetic stirrer at 1000 rpm for 30 min. Then, the required amount of epoxy resin was added to the HMWCNTs/ethanol mixture and stirred at 1000 rpm using a magnetic stirrer for 1 h. The resultant mixture was then mechanically stirred for 1 h and then ultrasonicated for an additional 1 h. The sonicated mixture was further mixed using a magnetic stirrer at 1000 rpm for 30 min at 80 °C. The purpose of such successive steps was to effectively break the agglomerates of HMWCNT and to completely evaporate the superfluous ethanol. A vacuum chamber was used for degassing the mixture for 30 min. Finally, the hardener was added and gently stirred until the homogeneity of the mixture was attained.

### 2.3. Preparation of HMWCNTs Composite Laminates

A dual action top-feed airbrush with a maximum pressure of 0.35 MPa and high flow rate air inlet valve was used to provide steady pressure during the spraying process. The viscosity of the mixture was maintained at an optimum range to avoid the deformation of the HMWCNTs. Then, the adjusted mixture of HMWCNTs/epoxy/hardener was sprayed onto the two interfaces of the CF/E prepregs using an airbrush.

**Figure 2:**
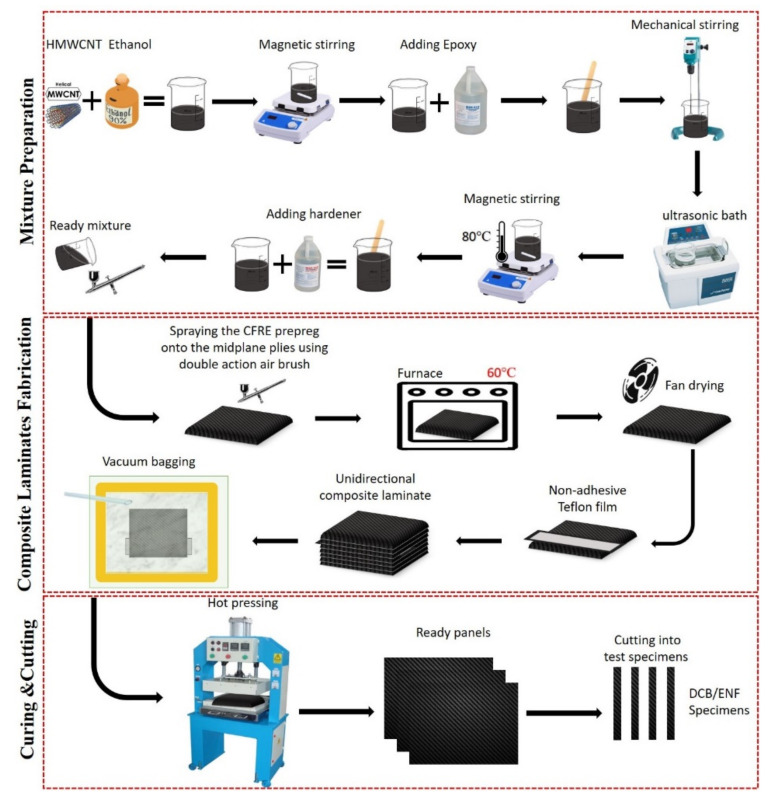
Schematic representation of the composite laminate preparation process flow.

Five steps were carefully followed to fabricate the composite laminates. First, a total of 16 unidirectional reinforced composite prepreg sheets measuring 300 mm by 230 mm were cut out from a frozen roll. Subsequently, an A3 paper was divided into equal sized rows and columns and the eighth and ninth epoxy/carbon fiber prepreg were placed on the middle of the paper. Using the dual action airbrush, the HMWCNTs/epoxy/hardener mixture was carefully spread over the two laminate surfaces forming a uniform coat. The resultant CF/E laminates were placed in an oven for 15 min at 60 °C. They were then removed from the oven and subjected to fan drying until all of the ethanol had completely evaporated and the mixture leftover on the laminates was well-dispersed. Secondly, seven layers of composite laminates (uncoated) were stacked up and then the above coated laminates were positioned on the top. A Teflon film with dimensions of 300 mm × 70 mm × 12 µm was placed in the middle plane of the eighth and ninth layers. The Teflon film was utilized to induce an initial edge crack in every sample. The rest of the layers were repeatedly stacked until the end. In the third step, the resultant composite panels were put under a vacuum in order to remove any entrapped air and to help consolidate the layup. For improved properties, the prepreg layups were debulked in a vacuum for 30 min. The fourth step comprised the composite laminates being placed in a hot press machine in accordance with the curing cycle by the manufacturer for curing, and then the composite panel was cooled down to room temperature. Finally, the naturally cooled composite laminate was cut into the desired dimension according to the ASTM standard for DCB and ENF testing and analysis. For each DCB test and analysis sample, piano hinges were attached adhesively at the location of the crack initiator and then the edges were painted with a correction fluid. In order to indicate the exact crack length, several vertical lines were drawn on the test samples. The steps are summarized in Figure 2.

### 2.4. Fracture Tests

#### 2.4.1. Double Cantilever Beam (DCB) Test

**Figure 3:**
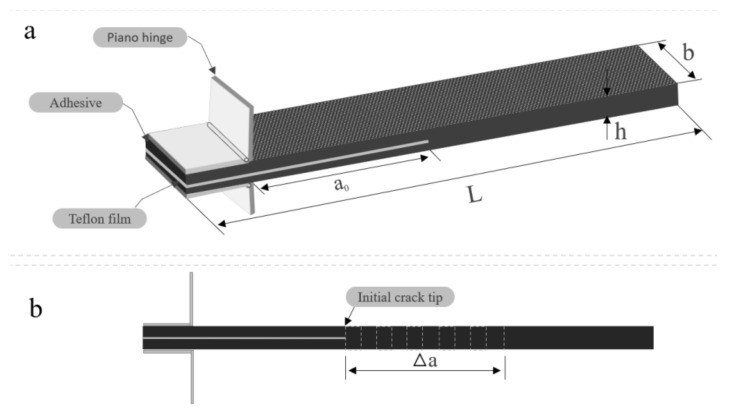
Schematic representation of the double cantilever beam (DCB) specimen: (**a**) with piano hinges; (**b**) initial crack tip

**Figure 4:**
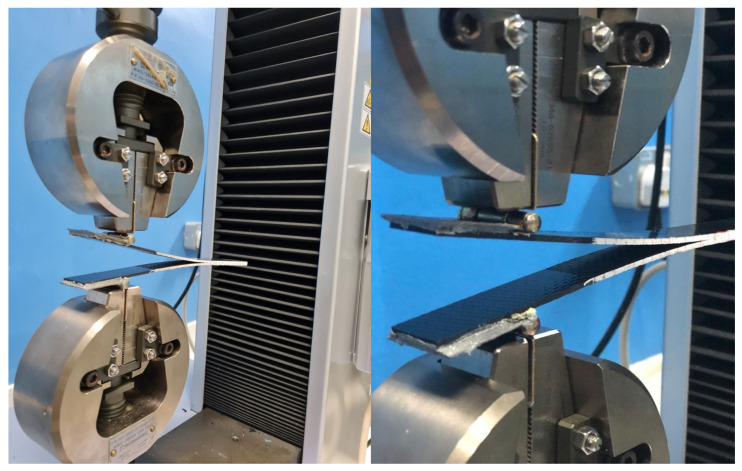
Experimental set up for the DCB specimen.

The interlaminar mode I fracture toughness (*G_IC_*) of the composite laminates was examined using a DCB test in a Shimadzu Autograph Precision Universal Testing Machine AG-Xplus Series (Shimadzu, Tokyo, Japan) equipped with a 10 kN load cell. The test procedure was conducted according to ASTM D5528 standard [66]. A schematic representation of the DCB test is shown in Figure 3, while the experimental set up for the DCB specimen is shown in Figure 4. The test sample had a 200 mm total length (L), 20 mm width (b), 50 mm initial crack length (a_0_), and 4.6 mm thickness (h), with a Teflon film of 12.7 μm thickness in their middle plane, as shown in Figure 3a. A set of five replicate specimens were employed for each test. One end of each specimen was painted with white correction liquid for the crack growth visualization during testing. Thin vertical lines were marked after every 5 mm from the initial crack tip, as shown in Figure 3b. This assisted in determining the changes in length. A displacement control mode was used to perform tests at a cross-head speed of 5 mm/min. During the test, the data recorded were load (P) versus displacement (δ). The sample compliance (C) was evaluated by the loading points displacement divided by the load applied. Three theories were applied to calculate *G_IC_*, and they are summarized in Table 1.

#### 2.4.2. End Notched Flexure (ENF) Test

**Figure 5:**
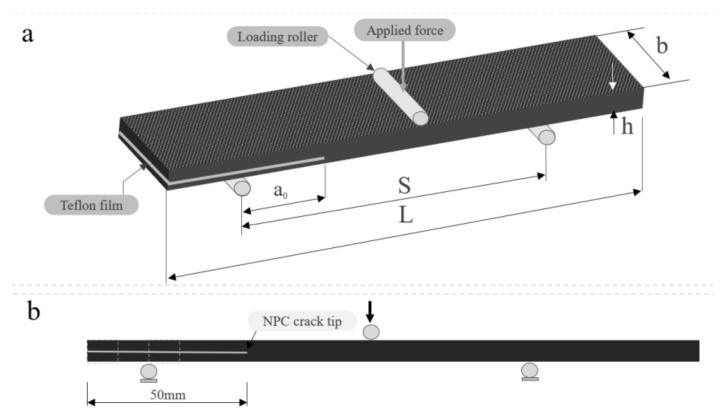
End notched flexure (ENF) specimen configuration: (**a**) specimen nomenclature; (**b**) crack tip location.

**Figure 6:**
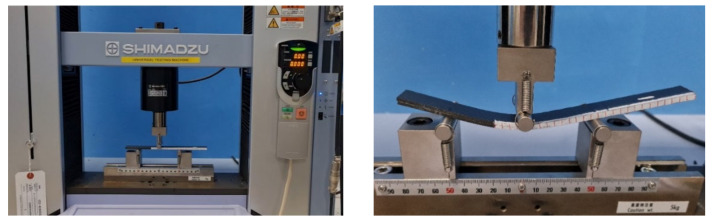
Experimental set up for the ENF specimen.

The mode II interlaminar fracture toughness (*G_IIC_*) of the composite laminate was examined with the ENF test method using Shimadzu Autograph Precision Universal Testing Machine AG-Xplus Series (Shimadzu, Tokyo, Japan) equipped with a 10 kN load cell. Figure 5 and Figure 6 illustrate the configuration of the ENF specimen and the experimental set up for the ENF specimen, respectively. The ENF test was performed with a three-point bending fixture that has loading roller and side supports. The performed tests were under the control of displacement at a cross-head speed of 1 mm/min. Tests were conducted for a crack length of 50 mm, where a peak load was applied until the crack propagated and the load started to drop. The ENF test sample measured 200 mm in length (L), 20 mm width (b), and 4.6 mm thickness (h). It had a span length (S) of 100 mm, in accordance with ASTM D7905 [67]. The results obtained with this method were the (*G_IIC_*) mode II interlaminar fracture toughness. The data of the load versus displacement were recorded to determine the mode II interlaminar fracture toughness, and *G_IIC_* was calculated using Equation (1) [68]:

(1) $$G_{IIC}=\frac{9a^{2}P^{2}}{16Eb^{2}h^{3}}$$

where *h* is the half specimen thickness, *P* is the maximum fracture test load of the corresponded crack length, *a* is the crack length, and *b* is the sample width.

All of the specimens for the respective tests are tabulated and shown in Table 2.

### 2.5. Field Emission Scanning Electron Microscopy (FESEM)

A Zeiss crossbeam 340 field emission scanning electron microscope (Carl Zeiss Microscopy GmbH, Jena, Germany) was used to study and analyze the fractured surfaces of the samples. The obtained FESEM images were then used to study the surface morphology and mechanisms of the fracture damage.

### 2.6. Thermogravimetric Analysis (TGA)

The thermal stability of the composite laminate was analyzed and determined using a thermo gravimetric analyzer (TGA) Q50 V20.13 Build 39 (TA Instruments, Haan, Germany). Three composite samples with different compositions were tested and analyzed. Each one was placed in an alumina crucible and subjected to a pyrolysis procedure in a nitrogen environment with a flow rate of 60 mL/min. The heating rate was 20 °C/min and the experiment was carried out from room temperature up to 800 °C.

### 2.7. Differential Scanning Calorimetry (DSC)

A DSC analyzer, DSC Q20 V24.11 Build 124 (Malvern Panalytical Ltd., Malvern, UK), was used to carry out the differential scanning calorimetry (DSC) testing. Three composite samples of different compositions (0 wt.%, 0.2 wt.%, and 0.4 wt.% of HMWCNT CF/E composite laminate) were tested and analyzed. Each sample was placed in an alumina crucible to effectively carry out the analysis. The laminates were tested at room temperature and progressively to elevated temperatures of 250 °C under N_2_ and O_2_ environments with a flow rate of 50 mL/min at a heating rate of 10 °C/min.

### 2.8. Electrical Conductivity

Volume resistivity test was carried out according to the ASTM F390-98 standard [69] and a four-point probe was employed to determine the volume resistivity of the composite laminate.

## 3. Results and Discussion

### 3.1. Mode I Interlaminar Fracture Toughness

**Figure 7:**
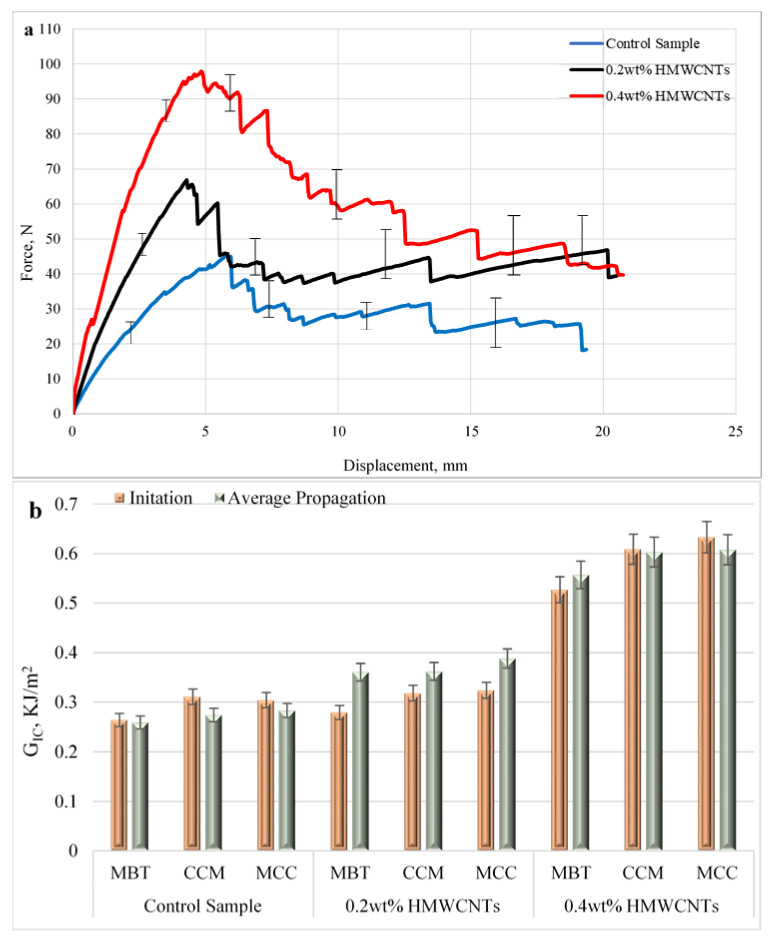
Mode I interlaminar fracture toughness: (**a**) average load versus displacement curves; (**b**) *G_IC_* values of the control, 0.2 wt.% and 0.4 wt.% HMWCNT composite samples.

Figure 7a depicts the average load versus displacement curves for the samples subjected to fracture tests, while Figure 7b represents the *G_IC_* values. Here, two *G_IC_* are defined—crack initiation and crack propagation. The *G_IC_* initiation is obtained using the theories given in Table 1, where the load (P) corresponds to the one at the onset of the linear deviation of the load–displacement curve. In the case of *G_IC_* propagation, the load (P) corresponds to the mean values attained at the plateau area. Both the *G_IC_* initiation and propagation are presented in Table 3. As seen in Figure 7a, the load increased in the linear form and gradually reached a critical value. When critical values were attained, the load suddenly tended to decrease to 50 N at a length of approximately 19 mm, which showed the initiation of delamination and propagation. All of the tests showed an unstable growth of delamination at the beginning that could be attributed to the artificial delamination caused by adding the Teflon layer in every sample in the middle plane. However, with the propagation of delamination along the interface of the middle plane, the load tends to decrease gradually along the interface till 19 mm, which indicates the stable growth of delamination. The fracture toughness increases with the incorporation of HMWCNT in comparison with the control sample. The maximum interlaminar fracture toughness *G_IC_* for the 0.2 wt.% and 0.4 wt.% HMWCNT was 0.36 kJ/m^2^ and 0.55 kJ/m^2^, respectively. The initiation and propagation values of mode I of the control, 0.2 wt.%, and 0.4 wt.% HMWCNT are depicted in Figure 7b and their respective values are presented in Table 3. The critical load value for the 0.2 wt.% HMWCNT composite sample was increased by 43.04%, while the 0.4 wt.% HMWCNT composite sample had greater improvements of 111.55%. It is clear that the composite laminates have a strong intermolecular bond and good dispersion quality between the epoxy and carbon nanotubes for the occurrence of load transfer. To explain this further, if the dispersion quality was poor, the carbon nanotubes would easily come out during delamination, and would therefore register a decrease in the maximum fracture toughness values that could be achieved [70]. Thus, it is clearly demonstrated from the experimental results and analysis that the interfacial chemical interaction between the epoxy and HMWCNTs resulted in an improved strength and fracture toughness determined by the dispersion technique. Similar conclusions have been made by other researchers; Saadati, et al. [71] reported a significant improvement in the fracture energies of mode I (*G_IC_*) and small increases in mode II (*G_IIC_*) in comparison with the plain system. They further affirmed that composites reinforced with CNTs have higher *G_IC_* values compared with those with unidirectional reinforcements.

### 3.2. Mode II Interlaminar Fracture Toughness

**Figure 8:**
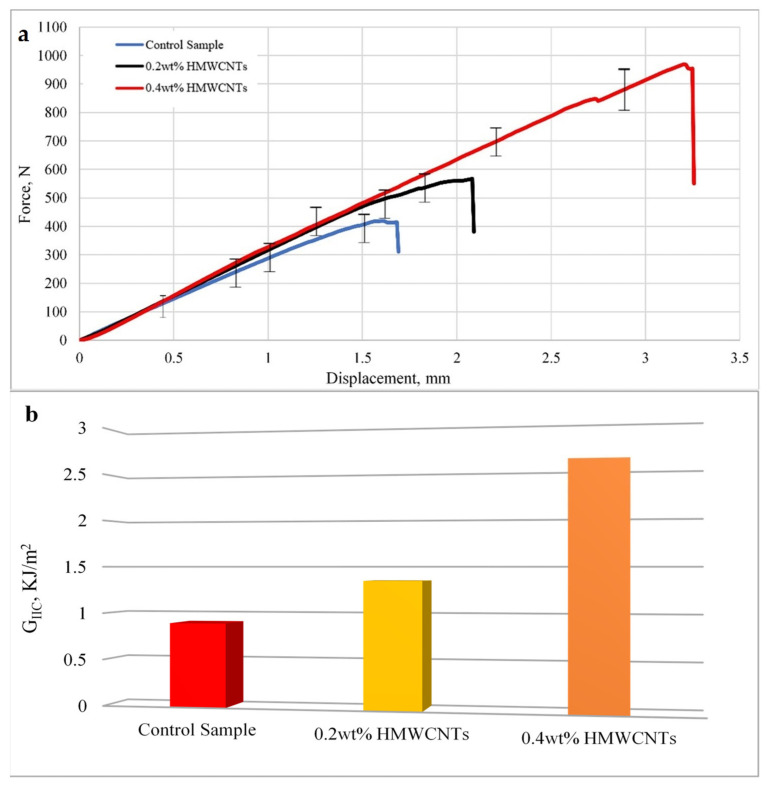
Mode II interlaminar fracture toughness results of the ENF test (**a**) Average load versus displacement curve; (**b**) *G_IIC_* values of control, 0.2 wt.%, and 0.4 wt.% HMWCNT composite samples.

Figure 8 shows the average load versus displacement curves in Figure 8a, and the values of *G_IIC_* are shown in Figure 8b. As shown in Figure 8a, an almost similar kind of behavior was observed for HMWCNTs with 0.2 wt.% and 0.4 wt.% composite laminates. The load applied increased linearly until the crack started to propagate, which is specified by the deviations observed in the elastic regions. The load versus displacement curve slope for the 0.2 wt.% HMWCNT sample almost coincided with the slope of the 0.4 wt.% HMWCNT composite laminate because of the addition of HMWCNTs. The higher content of HMWCNTs of 0.4 wt.% in the composite laminate led to a significant increase in load values. The load applied for all of the samples increased linearly and the crack propagation was determined at the point when the load started to drop. This is partly because of mid-plane plies sliding over each other when subjected to an in-plane shear loading, leading to unstable crack growth and a sudden load drop. The fracture toughness *G_IIC_* increased with the incorporation of HMWCNTs in comparison with the control sample. The maximum interlaminar fracture toughness *G_IIC_* observed for the 0.2 wt.% HMWCNT was 1.35 kJ/m^2^, while 2.59 kJ/m^2^ was noticed for the 0.4 wt.% HMWCNT. Figure 8b shows the fracture toughness values for mode II and the increase in the percentage of the fracture values by the control sample can be seen in Table 4. The fracture toughness in the 0.2 wt.% HMWCNT was increased to about 50.88%, while the higher amount of HMWCNT content 0.4 wt.% showed a significant improvement of 190%. The presented results for *G_IC_* and *G_IIC_* are in complete agreement that the addition of HMWCNT in CF/E composite laminates further promotes the interlaminar fracture toughness of modes I and II [71].

A comparison of the increased interlaminar fracture toughness for the modes I and II values obtained for the 0.2 wt.% and 0.4 wt.% HMWCNT composite laminates with the control sample are presented in Table 4. After analyzing the complete set of experimental data, it can be concluded that the addition of 0.2 wt.% and 0.4 wt.% HMWCNTs into the CF/E laminate increased the fracture toughness compared with the control sample. This improvement in fracture toughness is attributed to the dissipation mechanisms, which integrates into the structure of the material during the stage where crack propagation occurs. The reason behind energy absorption is the effect of the high fraction of HMWCNTs in a CF/E laminate. It can also be understood that the delamination in the control sample occurred because of a tensile crack in the polymer matrix related to the carbon fiber bridging, which resists the growth of delamination at the crack tip [72]. In the case of HMWCNTs, the same mechanism was observed, but another mechanism occurred with additional energy consumption, and the HMWCNTs also bridged the crack tip interface and resulted in an improved crack propagation resistance. The underlying mode I and mode II interlaminar fracture failure mechanism is highly influenced by the size of the fracture process zone (FPZ). Indeed, for HMWCNT enriched CF/epoxy resin composites, the size of the nonlinear FPZ emerging close to the tip of an interlaminar crack is by far more significant compared with the structure size. For higher a wt.% of HMWCNTs, discontinuous cracking, microcrack deflection, and pinning, or fiber/tow bridging of the crack, lead to highly nonlinear cohesive stresses, and hence the improved crack propagation resistance. Several researchers [73,74,75] have confirmed this underlying principle in the fracture of composite laminates. However, to capture and quantify the nonlinear stresses close to the crack tip, the introduction of a characteristic length scale associated with the size of the FPZ is necessary [73,74,75,76].

### 3.3. Fractographic Analysis

**Figure 9:**
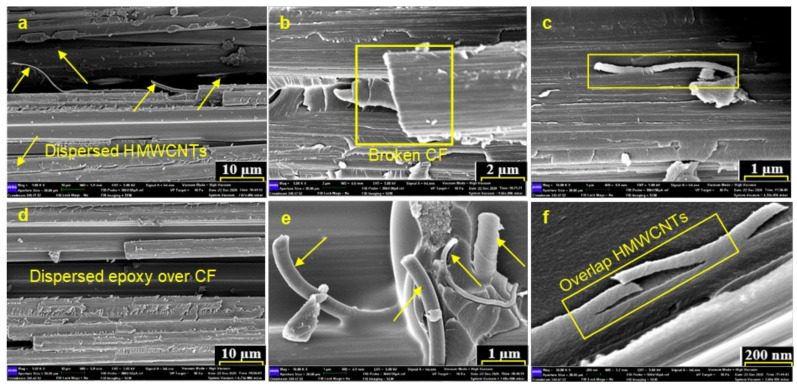
FESEM micrographs of the fractured surfaces (**a**–**c**) 0.2 wt.% and (**d**–**f**) 0.4 wt.% HMWCNT reinforced composites with visible HMWCNTs near fractured surface.

FESEM images at different magnifications of fractured surface after the DCB and ENF tests were taken are shown in Figure 8. The surface morphology of the fractured surface of 0.2 wt.% HMWCNT is illustrated in Figure 9a–c, and the fractured surface of 0.4 wt.% HMWCNT is represented in Figure 9d–f. From the morphology analysis, it is clear that increasing the wt.% amount of HMWCNTs in the composite laminate improves the total composite tensile and flexural strength, and in turn leads to a higher surface roughness. This affirms the embedment of HMWCNTs in the epoxy matrix along the crack interface predominantly in areas of a relatively smoother surface. Furthermore, the evidence of less HMWCNT agglomerates over the fracture surface was observed because of the optimal dispersion over the prepreg during the dispersion process. The bridging of HMWCNT and pulling-out at fractured zones of the composite laminates can be seen in Figure 9. It is attributed to the fact that the occurrence of a bridging mechanism is because of the better adhesion of the CF/E matrix and HMWCNTs, and thus the composite laminates have a significantly improved interlaminar fracture toughness.

### 3.4. Thermogravimetric Analysis (TGA)

**Figure 10:**
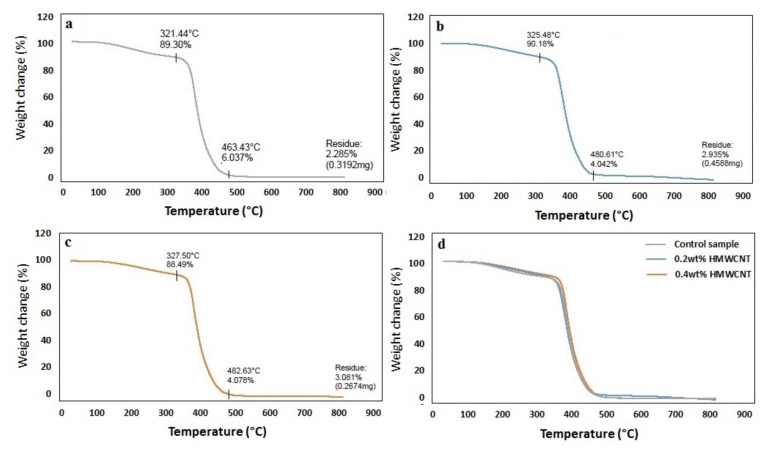
TGA analysis of the (**a**) control sample, the (**b**) 0.2 wt.% HMWCNT, the (**c**) 0.4 wt.% HMWCNT, and a (**d**) comparison of all of the specimens.

**Figure 11:**
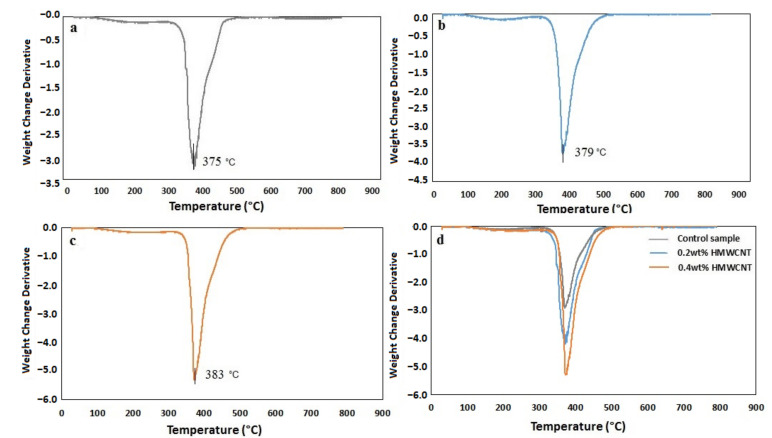
Derivative curves calculated from the TGA profiles of the (**a**) control sample, the (**b**) 0.2 wt.% HMWCNT, the (**c**) 0.4 wt.% HMWCNT, and (**d**) a comparison of all of the specimens.

**Figure 12:**
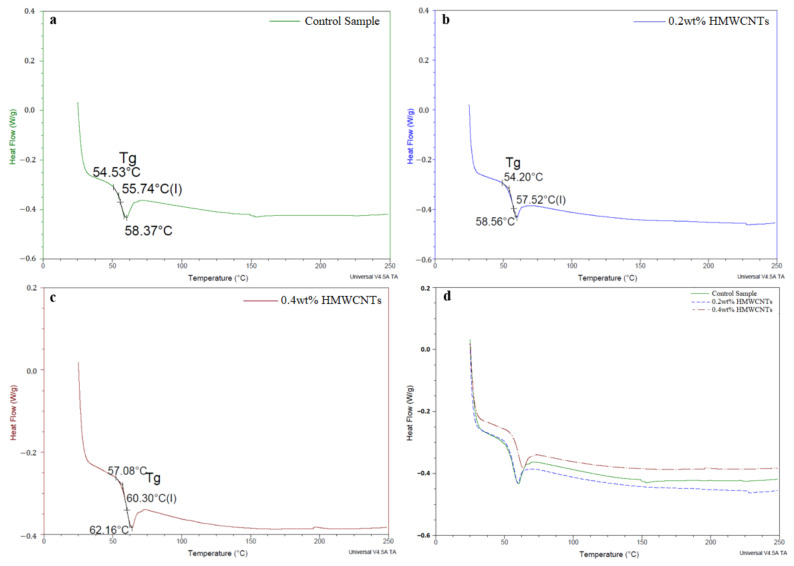
DSC analysis of the (**a**) control sample, the (**b**) 0.2 wt.% HMWCNT, the (**c**) 0.4 wt.% HMWCNT, and a (**d**) comparison of all of the specimens.

A TGA analysis was performed on the control sample and the two composite laminates with 0.2 wt.% HMWCNT and 0.4 wt.% HMWCNT. The results of this analysis are shown in Figure 10. An initial mass loss was observed for all of the tested samples, albeit with a slight difference regarding the highest degradation temperature of this initial mass loss event. The temperature increase from the control sample was 321.4 °C with the addition of CNTs, leading to 325.5 °C for the 0.2 wt.% HMWCNT and 327.5 °C for the 0.4 wt.% HMWCNT. The temperature rise contributed to the elimination of any moisture present and further caused the dehydration of the secondary alcoholic groups composing the epoxy material, and in turn, promoted the appearance of an unsaturated structure. This unsaturation process resulted in weak aliphatic C–O and C–N bonds. The second degradation (mass loss) phase occured at a temperature higher than 300 °C, specifically between 321–463 °C for the control, 325–481 °C for the 0.2 wt.% HMWCNT, and 327–483 °C for the 0.4 wt.% HMWCNT. The associated mass loss was related to the aromatic epoxy decomposition [77,78,79]. One last degradation phase, showing the degradation of laminates at the end of the aromatic degradation stage, occurred starting from 463 °C for the control sample (residual mass of 2.29 weight%), at 481 °C for the 0.2 wt.% HMWCNT (residual mass of 2.94 weight%), and at 483 °C for the 0.4 wt.% HMWCNT (residual mass of 3.09 wt.%). Regarding the CNTs, the residual mass is usually attributed to metal catalyst particles from the synthesis process [80]. This is further confirmed by the fact that the residual mass increases with an increase in the content of the CNTs in the samples, as more catalyst particles are introduced in the composites. In general, the samples stability appears to be strongly influenced by the CNT content, as indicated by the increasing degradation temperature in all of the observed decomposition events. This could likely be explained by the comparatively stronger bond between the epoxy and HMWCNT, which can delay the diffusion of small molecules from the resin matrix at high temperatures, thus resulting in an enhanced thermal stability [81]. In the past, some authors have reported a decrease in the decomposition temperature in epoxy composites by increasing the CNTs-to-resin ratio [81,82,83], which could be explained by the enhanced thermal conductivity of the composite upon the addition of the CNTs fraction. According to Figure 11, such an effect was not observed for the tested samples, meaning that the addition of CNTs in this study did not negatively affect the thermal stability of the composites, but rather imparted the opposite effect. The TGA performed in an oxidizing condition led to the decomposition of most carbon-based materials at around 650 °C, as shown in Figure 11 [79,84,85]. The derivative curves of the TGA (dW%/dt) were calculated and are shown in Figure 12. All of the plots show a very well-defined degradation peak at a temperature higher than 300 °C, which is indicative of the main degradative temperature for each material. The control sample structural disruption occurs at 375 °C, followed by the 0.2 wt.% HMWCNT at 379 °C and the 0.4 wt.% HMWCNT at 383 °C. This again implies that the addition of CNTs to the epoxy material is indeed related to increased thermal stability, even under strongly oxidizing conditions, as reported by many researchers in the literature [77,83,86].

### 3.5. Differential Scanning Calorimetry (DSC)

The DSC analysis was carried out for the control sample and the 0.2 wt.% and 0.4 wt.% HMWCNT composite laminates. This analysis was performed to evaluate the phase transition dynamics in the control sample and to compare it with the composite materials. The results are shown in Figure 12. The heat flow profiles exhibit a steep endothermic deviation at the very beginning of the experimental temperature window, which is strongly associated with the initial mass loss events occurring from 50 to 300 °C on all of the samples, as seen in Figure 10. The expansion work due to the compound evaporation is typically linked to a loss of heat at relatively low temperatures (usually below the temperature at which true degradation occurs). While epoxy resins are thermosetting materials, they still undergo a slight softening upon heating [87,88]. The endothermic peak shown in all of the plots is indicative of glass transition events, as one does not observe a related peak in the TGA curves, meaning that such an endothermic peak is not associated with mass loss, but rather with the fact that the material has reached a glass transition temperature. The *T*_g_ was identified as 55.74 °C for the control sample, 57.52 °C for the 0.2 wt.% HMWCNT composite, and 60.30 °C in the case of the 0.4 wt.% HMWCNT. This result is strong evidence that, upon the addition of a certain mass of CNTs, the *T*_g_ peak is shifted towards higher temperature. This is perfectly in line with the results reported by other authors regarding pure epoxy resins and their composite materials [82,84,89,90]. This temperature is affected by the change in the composition of the composite, as well as by the parameters such as temperature, time, heat load, and degree of orientation [86,88].

### 3.6. Electrical Conductivity

**Figure 13:**
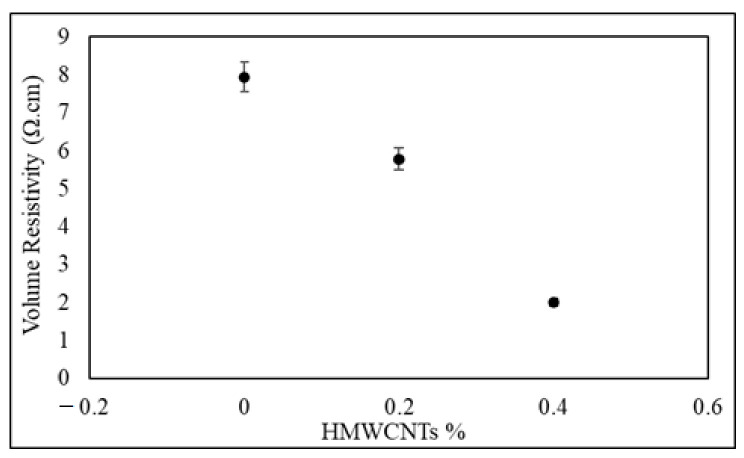
Volume resistivity of 0 wt.%, 0.2 wt.%, and 0.4 wt.% HMWCNTs.

The volume resistivity of the control and the 0.2 wt.% and 0.4 wt.% HMWCNTs are shown in Figure 13. The volume resistivity of the control, 0.2 wt.% HMWCNT, and 0.4 wt.% HMWCNT CF/E composite laminate samples was found to be 7.94 Ω.cm, 5.78 Ω.cm, and 2.01 Ω.cm, respectively. Indeed, the 0.4 wt.% HMWCNT CF/E composite laminate sample showed the lowest resistivity among all of the laminates. This is due to the increase in the amount of conductive CNT fillers in the insulating matrix. The different behavior in the electrical conductivity of composites laminates is in fact because of a different concentration of CNT. The fabrication method can cut the CNTs into smaller tubes, which results in a low porosity for the bucky papers, and a higher CNT concentration, which implies a greater conductivity improvement.

## 4. Conclusions

The HMWCNTs on unidirectional CF/E composite laminates were successfully fabricated using multi-dispersion techniques with different wt.% of filler loadings, and their effect on the interlaminar fracture toughness was investigated. The following conclusions were made:

i. Mode I fracture interlaminar toughness analysis by DCB showed a 39.15% improvement in fracture toughness for the 0.2 wt.% HMWCNT loading composite sample, while the higher loading of the 0.4 wt.% HMWCNT filler composite sample registered a remarkable increase in the fracture toughness by 115.05%. Mode II interlaminar fracture toughness analysis by ENF showed a 50.88% and 190% increase in the 0.2 wt.% and 0.4 wt.% HMWCNT composite laminates, respectively.
ii. The delamination in the control sample occurred because of the tensile crack in the matrix related to the carbon fiber bridging that resisted the growth of delamination at the crack tip. In the case of the HMWCNTs, the same mechanism was observed, but at the same time, another mechanism was noted with additional energy consumption, and the HMWCNTs also bridged the crack tip interface and resulted in improved crack propagation resistance.
iii. The FESEM analysis further confirmed the HMWCNT bridging and pull-out at the fracture zones of the CF/E, confirming the improved interlaminar fracture toughness. The TGA analysis showed that 0.4 wt.% HMWCNT laminates degraded 3 °C later than the 0.2 wt.% HMWCNT, generating a good bond between the epoxy and HMWCNT that could delay the diffusion of small molecules from the resin matrix at a high temperature, and therefore result in enhanced thermal stability.
iv. The DSC analysis show that the addition of HMWCNTs shifted the *T*_g_ to higher temperatures. The thermal analysis showed that by increasing the wt.% of the HMWNCTs, the thermal stability of the sample was further improved. The volume resistivity of the 0.4 wt.% HMWCNT showed the lowest resistivity among all the laminates because of the increase in the amount of conductive HMWCNTs in the insulating matrix.
v. Thus, this work presents an all-inclusive mode I and II inter- and intra-laminar fracture toughness, TGA and DSC analyses, and electrical conductivity on HMWCNTs-CF/E composite laminates, registering significant improvements in the respective properties. This is lacking in the existing works and, as such, the above experimental findings provide concrete evidence that the created interfacial intermolecular interaction between the epoxy and HMWCNT was strengthened by the dispersion technique that improved the fracture toughness. This has not only contributed immensely to the body of knowledge, but also constitutes a novel outcome that forms the basis for further research in this area.

## Figures and Tables

**Table 1:** *G_IC_* prediction using different theories [66].

Theories	Equation for Mode I Fracture Toughness	Symbols
Modified Beam Theory (MBT)	$G_{Ic}=\frac{3P\delta }{2b\left(a+\Delta \right)}$	*P* = Load, *δ* = Displacement, *b* = Width, *a* = Delamination length, *A*_1_ = Slope of plot of a/b versus *C*^1/3^, *n* = Slope of plot of Log c versus Log a, ∆ = Effective delamination extension to correct for the rotation of DCB arms at the delamination front
Compliance Calibration Method (CCM)	$G_{Ic}=\frac{nP\delta }{2ba}$	
Modified Compliance Calibration (MCC)	GIc=3P2C2/3 2A1bh	

**Table 2:** Tabulation of the specimens used in this study.

S/N	Performed Tests	Specimen Dimensions	Standard Used
1	Double Cantilever Beam (DCB)	200 mm length (L), 20 mm width (b), 50 mm initial crack length (a_0_) and 4.6 mm thickness (h) with Teflon film of 12 μm thickness at their middle plane.	ASTM D5528 [66]
2	End Notched Flexure (ENF)	200 mm length (L), 20 mm width (b), 50 mm initial crack length, and 4.6 mm thickness (h). Span length (S) of 100 mm.	ASTM D7905 [67]

**Table 3:** Summary of average mode I fracture toughness of the control, 0.2 wt.%, and 0.4 wt.% HMWCNT composite samples.

HMWCNTs Content (wt.%)	Interlaminar Fracture Toughness *G_IC_*, KJ/m^2^					
	Initiation			Average Propagation		
	MBT	CCM	MCC	MBT	CCM	MCC
Control	0.264	0.311	0.304	0.259	0.274	0.283
0.2 wt.% HMWCNTs	0.279	0.318	0.324	0.3604	0.362	0.388
0.4 wt.% HMWCNTs	0.527	0.609	0.633	0.557	0.603	0.608

**Table 4:** Mode I and mode II interlaminar fracture toughness improvements.

HMWCNTs Content (wt.%)	Interlaminar Fracture Toughness Increments for *G_IC_* & *G_IIC_*						
	*G_IC_* Initiation%			*G_IC_* Average Propagation%			*G_IIC_* %
	MBT	CCM	MCC	MBT	CCM	MCC	
0.2 wt.% HMWCNTs	5.68	2.25	6.57	39.15	32.11	37.10	50.88
0.4 wt.% HMWCNTs	99.62	95.81	108.22	115.05	120.07	114.84	190

## Data Availability

The data presented in this study are available on request from the corresponding author.
